# Vaginal Douching after Parturition

**Published:** 1897-10-23

**Authors:** 


### VAGINAL DOUCHING AFTER PARTURITION.

Dr. Peter Horrocks has done well in drawing the attention of the profession to what one might have imagined was an unnecessary proposition, namely, that routine douching of the vagina after parturition is not necessary. It might have been thought that the continued existence of the human race, to say nothing of a few other animals who have managed to continue their species without the aid of the instrument-maker, would have been sufficient to show that, whatever might be said in favour of the douche, at least it was not necessary; but the fact is that, so powerful is dogma, and so difficult is it for practitioners to escape the thraldom of what is "taught in the schools," that it has become necessary to prove and to defend even so elementary a proposition as this.

The public opinion which, in these matters, exercises a certain sway over the general practitioner, just as public opinion always exercises some sort of sway over everyone on whom it is brought to bear, is derived from and is fostered by the trained nurse; and the trained monthly nurse, who, happy being, is able to learn a more or less lucrative profession in three months (!), is apt to look upon the recipe which she has been taught for the conduct of a confinement as the only possible one. The monthly nurse, be it added, is trained in her art and mystery at a lying-in hospital, where all sorts of contrivances have to be kept in operation to counteract the evil consequences which are apt to ensue from the aggregation of a number of cases, and, among other things that she is taught, is the routine douche. Now, we all know what happens when the mistress interferes with the cook, and no doubt the practitioner who, so far as the success of his case goes, is absolutely in the nurse's hands, finds it difficult to interfere with a process like douching, which he knows the modern "monthly" looks upon as an absolutely essential part of her recipe. Dr. Horrocks, who speaks with authority, says that "recently trained midwifery nurses are so firmly impressed with the importance of syringing, that it is with difficulty that they can be prevented from carrying it out." The fact is, that the midwifery nurse is as proud of her syringe, by the use of which she feels herself distinguished from the Gamp, as the new hospital sister is of the particular pattern of her cap-strings, by which she is differentiated from the "pro," and it is no easy matter to persuade her to let syringing alone.

If, however, we bring our teachings to the test of nature and experience, we see that the female economy is perfectly capable of dealing with difficulties born within itself, that thousands of millions of creatures produce their young successfully without aid or interference, and that if the douche is necessary, it is only so in consequence of something which is done either by the nurse or accoucheur. The routine douche is, in fact, the corollary of the dirty finger, and it may be broadly stated that, if the external genitals of the patient and the hands of the doctor and the nurse are kept in a condition of surgical cleanliness, the douche is quite unnecessary.

Dr. Horrocks points out that in the Guy's Hospital lying-in charity, which is entirely external, the mortality from puerperal fever has been very low, although routine syringing has not been practised. The students, however, are strongly urged never to make a vaginal examination without first washing their hands and placing them in a solution of perchloride of mercury (1 in 1,000). "In 9,097 confinements in one of the lowest and poorest quarters of London, with bad hygienic surroundings, we get a mortality from septicæmia of ·06 per cent.;" notwithstanding that in many of these cases the child has been born before the arrival of the extern, and the woman has been assisted by the kindly-meant help of a neighbour. Good results then are to be had without the routine douche, even where it is impossible to secure the complete asepticism which can be obtained in hospitals. But Dr. Horrocks goes further than to show that the routine douche is unnecessary; he maintains that it is itself a cause of mischief, and that either from ignorance, or from inattention to detail, or from what one may call pure accident, a dirty syringe or water that is not aseptic not infrequently gets used and causes injury. The moral of his remarks is, then, to the effect that douching, useful as it is when it is required, does itself involve a certain risk; that its use is quite unnecessary unless septic matter has been introduced; and that such matter need not be introduced in ordinary cases if the usual precautions of antiseptic midwifery be observed.

